# Descriptive analysis of low versus elevated intracranial pressure on cerebral physiology in adult traumatic brain injury: a CENTER-TBI exploratory study

**DOI:** 10.1007/s00701-020-04485-5

**Published:** 2020-09-04

**Authors:** Frederick A. Zeiler, Ari Ercole, Manuel Cabeleira, Nino Stocchetti, Peter J. Hutchinson, Peter Smielewski, Marek Czosnyka, CENTER-TBI High Resolution (HR ICU) Sub-Study Participants and Investigators, Audny Anke, Ronny Beer, Bo-Michael Bellander, Erta Beqiri, Andras Buki, Manuel Cabeleira, Marco Carbonara, Arturo Chieregato, Giuseppe Citerio, Hans Clusmann, Endre Czeiter, Marek Czosnyka, Bart Depreitere, Ari Ercole, Shirin Frisvold, Raimund Helbok, Stefan Jankowski, Daniel Kondziella, Lars-Owe Koskinen, Ana Kowark, David K. Menon, Geert Meyfroidt, Kirsten Moeller, David Nelson, Anna Piippo-Karjalainen, Andreea Radoi, Arminas Ragauskas, Rahul Raj, Jonathan Rhodes, Saulius Rocka, Rolf Rossaint, Juan Sahuquillo, Oliver Sakowitz, Peter Smielewski, Nino Stocchetti, Nina Sundström, Riikka Takala, Tomas Tamosuitis, Olli Tenovuo, Andreas Unterberg, Peter Vajkoczy, Alessia Vargiolu, Rimantas Vilcinis, Stefan Wolf, Alexander Younsi, Frederick A. Zeiler

**Affiliations:** 1 Department of Physical Medicine and Rehabilitation, University hospital Northern Norway, Tromsø, Norway; 2 Department of Neurology, Neurological Intensive Care Unit, Medical University of Innsbruck, Innsbruck, Austria; 3 Department of Neurosurgery & Anesthesia & intensive care medicine, Karolinska University Hospital, Stockholm, Sweden; 4 NeuroIntensive Care, Niguarda Hospital, Milan, Italy; 5 Department of Neurosurgery, Medical School, University of Pécs, Hungary and Neurotrauma Research Group, János Szentágothai Research Centre, University of Pécs, Pécs, Hungary; 6 Brain Physics Lab, Division of Neurosurgery, Dept of Clinical Neurosciences, University of Cambridge, Addenbrooke’s Hospital, Cambridge, UK; 7 Neuro ICU, Fondazione IRCCS Cà Granda Ospedale Maggiore Policlinico, Milan, Italy; 8 NeuroIntensive Care Unit, Department of Anesthesia & Intensive Care, ASST di Monza, Monza, Italy; 9 School of Medicine and Surgery, Università Milano Bicocca, Milano, Italy; 10 Department of Neurosurgery, Medical Faculty RWTH Aachen University, Aachen, Germany; 11 Department of Neurosurgery, University of Pecs and MTA-PTE Clinical Neuroscience MR Research Group and Janos Szentagothai Research Centre, Hungarian Brain Research Program (Grant No. KTIA 13 NAP-A-II/8), University of Pecs, Pecs, Hungary; 12 Department of Neurosurgery, University Hospitals Leuven, Leuven, Belgium; 13 Division of Anaesthesia, University of Cambridge, Addenbrooke’s Hospital, Cambridge, UK; 14 Department of Anesthesiology and Intensive care, University Hospital Northern Norway, Tromso, Norway; 15 Neurointensive Care, Sheffield Teaching Hospitals NHS Foundation Trust, Sheffield, UK; 16 Departments of Neurology, Clinical Neurophysiology and Neuroanesthesiology, Region Hovedstaden Rigshospitalet, Copenhagen, Denmark; 17 Department of Clinical Neuroscience, Neurosurgery, Umeå University, Umeå, Sweden; 18 Department of Anaesthesiology, University Hospital of Aachen, Aachen, Germany; 19 Intensive Care Medicine, University Hospitals Leuven, Leuven, Belgium; 20 Department Neuroanesthesiology, Region Hovedstaden Rigshospitalet, Copenhagen, Denmark; 21 Helsinki University Central Hospital, Helsinki, Finland; 22 Department of Neurosurgery, Vall d’Hebron University Hospital, Barcelona, Spain; 23 Department of Neurosurgery, Kaunas University of technology and Vilnius University, Vilnius, Lithuania; 24 Department of Anaesthesia, Critical Care & Pain Medicine NHS Lothian & University of Edinburg, Edinburgh, UK; 25 Klinik für Neurochirurgie, Klinikum Ludwigsburg, Ludwigsburg, Germany; 26 Department of Neurosurgery, University Hospital Heidelberg, Heidelberg, Germany; 27 Department of Pathophysiology and Transplantation, Milan University, and Neuroscience ICU, Fondazione IRCCS Cà Granda Ospedale Maggiore Policlinico, Milano, Italy; 28 Department of Radiation Sciences, Biomedical Engineering, Umea University, Umea, Sweden; 29 Perioperative Services, Intensive Care Medicine, and Pain Management, Turku University Central Hospital and University of Turku, Turku, Finland; 30 Neuro-intensive Care Unit, Kaunas University of Health Sciences, Kaunas, Lithuania; 31 Rehabilitation and Brain Trauma, Turku University Central Hospital and University of Turku, Turku, Finland; 32 Neurologie, Neurochirurgie und Psychiatrie, Charité – Universitätsmedizin Berlin, Berlin, Germany; 33 Department of Neurosurgery, Kaunas University of Health Sciences, Kaunas, Lithuania; 34 Department of Neurosurgery, Charité – Universitätsmedizin Berlin, corporate member of Freie Universität Berlin, Humboldt-Universität zu Berlin, and Berlin Institute of Health, Berlin, Germany; 35 Section of Neurosurgery, Department of Surgery, Rady Faculty of Health Sciences, University of Manitoba, Winnipeg, Canada

**Keywords:** Cerebral physiology, Cerebrovascular reactivity, ICP extremes, Outcomes

## Abstract

**Background:**

To date, the cerebral physiologic consequences of persistently elevated intracranial pressure (ICP) have been based on either low-resolution physiologic data or retrospective high-frequency data from single centers. The goal of this study was to provide a descriptive multi-center analysis of the cerebral physiologic consequences of ICP, comparing those with normal ICP to those with elevated ICP.

**Methods:**

The Collaborative European NeuroTrauma Effectiveness Research in Traumatic Brain Injury (CENTER-TBI) High-Resolution Intensive Care Unit (HR-ICU) sub-study cohort was utilized. The first 3 days of physiologic recording were analyzed, evaluating and comparing those patients with mean ICP < 15 mmHg versus those with mean ICP > 20 mmHg. Various cerebral physiologic parameters were derived and evaluated, including ICP, brain tissue oxygen (PbtO_2_), cerebral perfusion pressure (CPP), pulse amplitude of ICP (AMP), cerebrovascular reactivity, and cerebral compensatory reserve. The percentage time and dose above/below thresholds were also assessed. Basic descriptive statistics were employed in comparing the two cohorts.

**Results:**

185 patients were included, with 157 displaying a mean ICP below 15 mmHg and 28 having a mean ICP above 20 mmHg. For admission demographics, only admission Marshall and Rotterdam CT scores were statistically different between groups (*p* = 0.017 and *p* = 0.030, respectively). The high ICP group displayed statistically worse CPP, PbtO_2_, cerebrovascular reactivity, and compensatory reserve. The high ICP group displayed worse 6-month mortality (*p* < 0.0001) and poor outcome (*p* = 0.014), based on the Extended Glasgow Outcome Score.

**Conclusions:**

Low versus high ICP during the first 72 h after moderate/severe TBI is associated with significant disparities in CPP, AMP, cerebrovascular reactivity, cerebral compensatory reserve, and brain tissue oxygenation metrics. Such ICP extremes appear to be strongly related to 6-month patient outcomes, in keeping with previous literature. This work provides multi-center validation for previously described single-center retrospective results.

## Introduction

Intracranial pressure (ICP) has long been the focus of the critical care management in moderate/severe traumatic brain injury (TBI) patients. Consensus-based treatment guidelines in TBI care, such as those from the Brain Trauma Foundation (BTF), have gone through various renditions over the years, with shifting focus on ICP and cerebral perfusion pressure (CPP) thresholds [6]. Current guidelines suggest considering an ICP threshold for treatment at 20 or 22.5 mmHg while maintaining CPP between 60 and 70 mmHg [6, 16].

Various retrospective studies over the past few decades have sought to describe cerebral physiologic phenomena of elevations in ICP [1, 3, 8, 9, 11, 14]. Data from such preliminary work supports the association between persistently elevated ICP and mortality at 6 months post-TBI. [6, 14, 24] Similarly, persistent ICP elevations have been shown to be potentially linked to lower CPP values, [6] worse compensatory reserve, [5, 34] impaired cerebrovascular reactivity, [1, 10, 14] lower PbtO_2_ values, [16, 22] and autonomic dysfunction, [15, 26] in those rare data sets with high-frequency digital physiology. However, most studies have suffered from several limitations. One major criticism is that such work has arisen from retrospectively processed data, obtained and published from only a few (or single) centers with specialty expertise in biomedical signal analytic techniques in TBI.

The European Union–based Collaborative European NeuroTrauma Effectiveness Research in Traumatic Brain Injury (CENTER-TBI) study has aimed to produce a unique prospectively collected multi-center international TBI data set [20]. The High-Resolution Intensive Care Unit (HR-ICU) Sub-Study of CENTER-TBI has led to the creation of a unique high-frequency physiologic data set for moderate/severe TBI patients, allowing for a prospective multi-center validation/exploration of previous single-center retrospective findings. The goal of this specific study was to provide a descriptive multi-center analysis of the cerebral physiologic consequences of normal or elevated ICP, comparing those with mean ICP values below 15 mmHg to those with mean ICP values above 20 mmHg.

## Methods

### Patient population

All patients from the multi-center CENTER-TBI high-resolution ICU monitoring cohort with parenchymal ICP monitoring were included in this analysis. We further selected only patients with a mean ICP value in the first 3 days of recording either below 15 mmHg, or above 20 mmHg. These two cohorts were selected so as to focus on the cohorts of patients with grossly normal and abnormal ICP, respectively.

Patients with EVD based ICP data were excluded given the interrupted nature of their recordings. All patients were prospectively recruited between January 2015 and December 2017 from 21 centers in the European Union (EU). All patients were admitted to ICU for their TBI during the course of the study, with high-frequency digital signals recorded from their ICU monitors during the course of their ICU stay. All patients suffered predominantly from moderate to severe TBI (moderate = Glasgow Coma Score (GCS) 9 to 12, and severe = GCS of 8 or less). A minority of patients were categorized at the time of admission as suffering from less severe TBI, but experienced subsequent early deterioration leading to ICU admission for care and monitoring. All patients in this cohort had invasive ICP monitoring conducted in accordance with the BTF guidelines [6].

### Ethics

Data used in these analyses were collected as part of the CENTER-TBI study which had individual national or local regulatory approval; the UK Ethics approval is provided as an exemplar: (IRAS No: 150943; REC 14/SC/1370). The CENTER-TBI study (EC grant 602150) has been conducted in accordance with all relevant laws of the EU if directly applicable or of direct effect and all relevant laws of the country where the Recruiting sites were located including, but not limited to, the relevant privacy and data protection laws and regulations (the “Privacy Law”), the relevant laws and regulations on the use of human materials, and all relevant guidance relating to clinical studies from time to time in force including, but not limited to, the ICH Harmonized Tripartite Guideline for Good Clinical Practice (CPMP/ICH/135/95) (“ICH GCP”) and the World Medical Association Declaration of Helsinki entitled “Ethical Principles for Medical Research Involving Human Subjects”. Informed consent by the patients and/or the legal representative/next of kin was obtained, accordingly to the local legislations, for all patients recruited in the Core Dataset of CENTER-TBI and documented in the e-CRF.

### Data collection

As part of recruitment to the multi-center high-resolution ICU cohort of CENTER-TBI, all patients had demographics and injury data prospectively recorded. Similarly, all patients had high-frequency digital signals from ICU monitoring recorded throughout their ICU stay, with the goal of initiating recording within 24 h of ICU admission. All digital ICU signals were further processed (see Signal acquisition/Signal processing). For the purpose of providing a description of the population for this study, basic admission demographics and centrally reported CT variables for the first available CT of each patient were extracted [23]. They included age, admission best GCS motor score and pupillary reactivity (bilaterally reactive, unilateral reactive, bilateral unreactive), Marshall CT Classification, [21] Rotterdam CT score, [19] Helsinki CT Score, [23] presence or absence of traumatic subarachnoid hemorrhage (tSAH), extradural hematoma (EDH), pre-hospital hypotension, and pre-hospital hypoxia. CENTER-TBI data version 2.1 was accessed for the purpose of this study, via Opal database software [13].

### Signal acquisition

Arterial blood pressure (ABP) was obtained through arterial lines connected to pressure transducers. ICP was acquired from an intra-parenchymal strain gauge probe (Codman ICP MicroSensor; Codman & Shurtleff Inc., Raynham, MA) and parenchymal fiber optic pressure sensor (Camino ICP Monitor; Integra Life Sciences, Plainsboro, NJ, United States; https://www.integralife.com/). PbtO_2_ monitoring occurred via invasive parenchymal monitoring (Licox probe; Integra, Licox Brain Oxygen Monitoring System, Plainboro, NJ), typically placed in the frontal lobe. All signals were recorded using digital data transfer or digitized via an A/D converter (DT9803; Data Translation, Marlboro, MA), where appropriate; sampled at frequency of 100 Hz (Hz) or higher, using the ICM+ software (Cambridge Enterprise Ltd., Cambridge, UK, http://icmplus.neurosurg.cam.ac.uk) or Moberg CNS Monitor (Moberg Research Inc., Ambler, PA, USA, https://www.moberg.com) or a combination of both. Signal artifacts were removed using both manual and automated methods prior to further processing or analysis.

### Signal processing

Post-acquisition processing of the aforementioned signals was conducted using ICM+ (Cambridge Enterprise Ltd., Cambridge, UK, http://icmplus.neurosurg.cam.ac.uk). CPP was determined using the formula: CPP = MAP − ICP. Pulse amplitude of ICP (AMP) was determined by calculating fundamental Fourier amplitude of the ICP pulse waveforms over a 10-s window, updated every 10 s. Ten-second moving averages (updated every 10 s to avoid data overlap) were calculated for all recorded signals: ICP, ABP (which produced MAP), AMP, CPP, and PbtO_2_. This moving average filter was applied to decimate the raw signals to a frequency range association with the slow-wave vasogenic response.

We then derived ICP-based measures of cerebrovascular reactivity, using the Pearson correlation between 30 consecutive 10-s mean values of recorded physiology, updated every minute. PRx was derived as the correlation between ICP and MAP [12]. Pulse amplitude index (PAx) was derived as the correlation between AMP and MAP [2]. Finally, RAC was derived as the correlation (R) between AMP (A) and CPP (C) [29]. RAP, an index of cerebral compensatory reserve, was also derived as the correlation (R) between AMP (A) and ICP (P) [5, 18].

Data were output in minute-by-minute update frequency for the entire recording period. We then limited the data for analysis to the first 3 days of recording in order to focus on the acute phase commonly associated with cerebral physiologic derangements. Finally, we filtered out patients with mean ICP over the first 3 days of recording between 15 and 20 mmHg, so as to focus on comparing cerebral physiology between those with mean ICP < 15 mmHg, and those with mean ICP > 20 mmHg. All data curation and processing occurred in R (R Core Team (2019). R: A language and environment for statistical computing. R Foundation for Statistical Computing, Vienna, Austria. URL https://www.R-project.org/). Grand summary data for each patient, for the first 3 days of recording, were produced as follows:

1. *Grand average values*: ICP, CPP, MAP, AMP, PbtO_2_, PRx, PAx, RAC, RAP
2. *% Time above/below CPP thresholds*: We determined the percentage of time spent above BTF defined CPP threshold of 70 mmHg, and below the threshold of 60 mmHg. [6]
3. *% Time below PbtO*_*2*_ *threshold of 20 mmHg*. [22]
4. *% Time above cerebrovascular reactivity critical thresholds*: We determined the percentage of time spent above the following literature-defined PRx, PAx, and RAC thresholds: [24, 31]
  - PRx: 0, + 0.25, + 0.35
  - PAx: 0, + 0.25
  - RAC: − 0.10, − 0.05
5. *Mean hourly dose below PbtO*_*2*_ *of 20 mmHg.*
6. *Mean hourly dose above cerebrovascular reactivity critical threshold:* See above for literature-defined critical threshold utilized.
7. *Area under RAP over time:* Given complexities with interpreting RAP, we determined the area under the RAP over time curve for the RAP thresholds of 0 and + 0.4 (RAP AUC 0, and RAP AUC + 0.4), using a linear interpolation method of integration. Such methodology has been described previously by our group [34, 35].

### Statistical analysis

All statistical analysis was performed using R statistical computing software. The focus of this study was to provide a descriptive exploratory analysis of differences in cerebral physiology between patients with low mean ICP (i.e., ICP < 15 mmHg) and those with persistently high ICP (i.e., mean ICP > 20 mmHg). For continuous variables, Shapiro–Wilks test was used to test for normality. For all statistical testing performed, alpha was set at 0.05, with no corrections for multiple comparisons undertaken given the exploratory nature of this study.

Patient characteristics were summarized using mean, median, standard deviation, and inter-quartile range (IQR), where appropriate. Box plots and histograms were produced to highlight differences in characteristics and cerebral physiology, between the < 15 and > 20 mmHg cohorts. Similarly, Mann–Whitney *U* and *χ*^2^ testing were utilized to compare differences between these two cohorts, where applicable.

Finally, we briefly assessed differences in dichotomized 6-month outcomes, using *χ*^2^ testing. For this, GOSE was dichotomized into both alive/dead, and favorable/unfavorable (favorable = lower moderate disability or better, unfavorable = upper severe disability or worse). Further, to confirm the independent association between ICP and outcome, we evaluated the association between ICP and dichotomized outcome using multi-variable logistic regression models, adjusting for admission characteristics (i.e., age, admission GCS motor score, pupillary response, Marshall CT grade, and pre-hospital hypoxia/hypotension). For such models, we reported the area under the receiver operating curve (AUC), 95% confidence intervals (CI), and *p* value. AUC and 95% CIs were derived using a bootstrap process with 2000 iterations.

## Results

### Patient population characteristics

High-resolution physiologic recordings were available from 225 non-EVD patients. A total of 185 patients met the inclusion criteria for this study from the CENTER-TBI HR-ICU sub-study, with 157 displaying a mean ICP less than 15 mmHg and 28 with a mean ICP above 20 mmHg, during the first 72 h of recording. For the group with ICP below 15 mmHg, the median age was 51 years (IQR 31 to 62.3 years), median admission GCS of 6 (IQR 3 to 7), and 122 males (77.7%). Similarly, the group with ICP above 20 mmHg had a median age of 54 years (IQR 35.3 to 68.3 years), median admission GCS of 7 (IQR 3 to 8), and 19 males (67.9%). Table 1 provides a detailed outline of the patient demographics and admission characteristics for both cohorts. Of note, Marshall and Rotterdam CT scores on admission were statistically worse in the ICP above 20 mmHg group (*p* = 0.017 and *p* = 0.030, respectively).

**Table 1:** Patient admission demographics, injury and outcome data—Mann–Whitney *U*/*χ*^2^ testing—ICP below 15 mmHg versus ICP above 20 mmHg

**Demographic variable**		**Mean ICP below 15 mmHg**		**Mean ICP Above 20 mmHg**		**Mann–Whitney** ***U*/*χ***^**2**^ ***p*** **value**
		***N*** **= 157**		***N*** **= 28**		
		*Median*	*IQR*	*Median*	*IQR*	
Age (years)		51	31–62.3	54	35.3–68.3	0.311
Sex (male)		122	NA	19	NA	0.385
Admission total GCS		6	3–7	7	3–8	0.611
Admission GCS motor		4	1–5	3	2–4	0.263
Pupillary response	0	117	NA	18	NA	NS**
	1	13		2		
	2	27		8		
Pre-hospital hypoxia		21	NA	7	NA	0.201
Pre-hospital hypotension		22	NA	1	NA	0.215
Admission Marshall CT grade		3	2–6	6	3–6	**0.017**
Admission Rotterdam CT grade		3	3–4	4	3–5	**0.030**
Admission Helsinki CT grade		4	2–6	6	3–9	0.149
Epidural lesion		29	NA	7	NA	0.240
Traumatic subarachnoid hemorrhage		100	NA	14	NA	0.900
Alive at 6 months*		119	NA	9	NA	**<0.0001**
Favorable outcome at 6 months*		64	NA	5	NA	**0.014**

Favorable outcome = based on dichotomized 6-month Glasgow Outcome Score Extended (favorable = 5 or higher, unfavorable = 4 or less), GCS = Glasgow Coma Scale, IQR = interquartile range, *N* = number of patients, NA = not applicable. Note: pupillary response graded according to 0 = bilaterally reactive, 1 = unilateral unreactive, 2 = bilaterally unreactive; *p* values reaching statistical significance (< 0.05) are bolded

*20 patients did not have a 6-month outcome available

**All *χ*^2^ testing between various groupings of pupillary response failed to reach statistical significance

### Cerebral physiological consequences: ICP below 15 mmHg vs. ICP above 20 mmHg

**Fig. 1:**
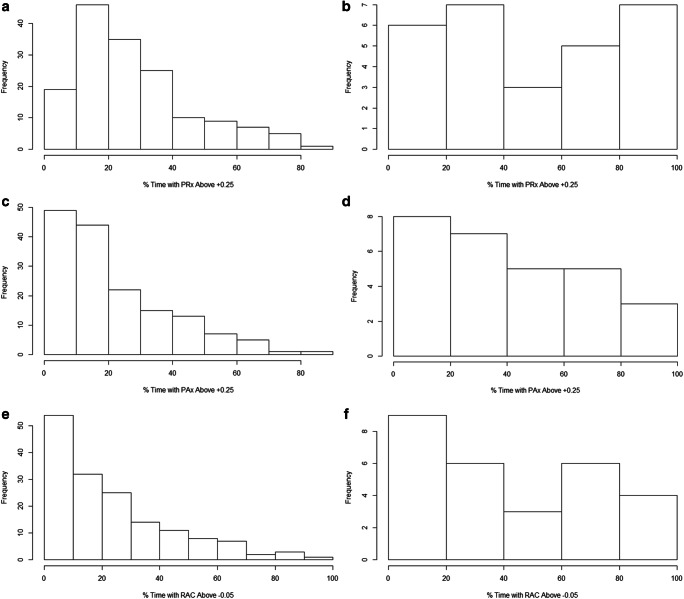
Histograms for % time above cerebrovascular reactivity threshold. AMP = pulse amplitude index, CPP = cerebral perfusion pressure, Frequency = number of patients, ICP = intracranial pressure, MAP = mean arterial pressure, PAx = pulse amplitude index (correlation between AMP and MAP), PRx = pressure reactivity index (correlation between ICP and MAP), RAC = correlation between AMP and CPP. **a** Histogram of % time with PRx above + 0.25–ICP < 15 mmHg group. **b** Histogram of % time with PRx above + 0.25–ICP > 20 mmHg group. **c** Histogram of % time with PAx above +0.25–ICP < 15 mmHg group. **d** Histogram of % time with PAx above + 0.25–ICP > 20 mmHg group. **e** Histogram of % time with RAC above − 0.05–ICP < 15 mmHg group. **f** Histogram of % time with RAC above − 0.05–ICP > 20 mmHg group

Evaluating differences in cerebral physiologic responses between the two ICP non-overlapping cohorts, various patterns were seen. Table 2 highlights all cerebral physiologic measures between both groups, and the results of Mann–Whitney *U* testing. The high ICP cohort displayed statistically significant higher MAP (*p* = 0.0009), lower CPP (*p* < 0.0001), and higher AMP (*p* < 0.0001). Mean PbtO_2_ was not different between the two groups, though the mean hourly dose of PbtO_2_ below 20 mmHg was significantly higher in the elevated ICP group (*p* = 0.033). Cerebrovascular reactivity, measured through PRx/PAx/RAC metrics, displayed statistically higher mean (*p* < 0.004 for all), percentage of time above critical threshold (*p* < 0.004 for all), and mean hourly dose above critical threshold (*p* < 0.004 for all). Similarly, worse compensatory reserve was also seen in the high ICP group, with trends toward smaller integrated RAP AUC values. Figure 1 provides histogram representation of the percentage of time above cerebrovascular reactivity thresholds for both groups.

**Table 2:** Cerebral physiologic data—Mann–Whitney *U* testing—ICP below 15 mmHg versus ICP above 20 mmHg

**Demographic variable**	**Mean ICP below 15 mmHg**		**Mean ICP above 20 mmHg**		**Mann–Whitney** ***U*** ***p*** **value**
	***N*** **= 157**		***N*** **= 28**		
	Median	IQR	Median	IQR	
ICP (mmHg)	11.0	8.2–12.9	23.4	21.9–37.3	**<0.0001**
MAP (mmHg)	82.0	76.2–87.5	89	80.2–96.8	**0.0009**
CPP (mmHg)	71.8	66.2–77.6	59.1	50.0–73.1	**<0.0001**
AMP (mmHg)	1.9	1.4–2.7	3.5	2.2–5.6	**<0.0001**
PbtO_2_ (mmHg)*	27.0	23.2–33.1	22.1	18.2–26.2	0.183
PRx (a.u.)	− 0.002	− 0.118 to 0.014	0.206	− 0.009 to 0.582	**0.0006**
PAx (a.u.)	− 0.090	− 0.208 to 0.095	0.151	− 0.090 to 0.376	**0.0001**
RAC (a.u.)	− 0.384	− 0.554 to − 0.176	− 0.181	− 0.382 to 0.145	**0.003**
RAP (a.u.)	0.731	0.560–0.837	0.710	0.485–0.783	0.290
RAP AUC 0	2662.2	1908.0–3369.3	2194.0	1000.0–3198.9	**0.047**
RAP AUC + 0.4	1305.6	716.4–1764.6	964.0	245–1565.8	0.052
Total length of recording (h)	119.7	80.5–161.6	114.1	57.1–167.7	0.333
% Time above/below threshold					
Below CPP 60 mmHg	7.3	2.2–26.3	57.2	12.4–75.4	**<0.0001**
Above CPP 70 mmHg	53.0	30.6–81.2	12.7	3.5–55.5	**0.0001**
Above PRx 0	47.8	22.6–64.3	72.4	47.1–90.2	**0.0009**
Above PRx + 0.25	23.7	14.0–38.0	46.1	26.2–82.1	**0.0003**
Above PRx + 0.35	17.0	9.7–28.0	36.4	18.3–77.9	**0.0003**
Above PAx 0	39.6	25.6–59.9	66.4	41.4–79.6	**0.0002**
Above PAx + 0.25	16.0	8.8–32.1	36.4	19.2–62.6	**<0.0001**
Above RAC − 0.10	20.9	9.2–40.4	39.4	18.1–75.1	**0.003**
Above RAC − 0.05	17.6	7.8–35.4	35.3	15.9–71.2	**0.002**
Below PbtO_2_ 20 mmHg*	16.5	1.7–35.1	30.7	19.3–54.3	0.229
Mean hourly dose above/below threshold					
Above PRx 0	7.5	4.9–11.3	12.5	6.2–26.9	**0.003**
Above PRx + 0.25	2.9	1.7–4.4	6.0	2.5–16.1	**0.0006**
Above PRx + 0.35	1.8	1.1–2.9	4.1	1.6–12.3	**0.0004**
Above PAx 0	5.0	2.9–9.5	10.3	5.9–17.3	**0.0003**
Above PAx + 0.25	1.6	0.7–3.4	4.3	2.3–8.6	**<0.0001**
Above RAC − 0.10	2.6	1.1–5.4	5.8	2.1–15.8	**0.001**
Above RAC − 0.05	2.0	0.8–4.0	4.9	1.8–13.7	**0.0007**
Below PbtO_2_ 20 mmHg*	3.1	0.3–55.2	94.8	49.9–144.1	**0.033**

*p* values reaching statistical significance (<0.05) are bolded

a.u. = arbitrary units, AUC = integrated area under the RAP over time plot, AMP = pulse amplitude of ICP, CPP = cerebral perfusion pressure, ICP = intracranial pressure, IQR = interquartile range, MAP = mean arterial pressure, mmHg = millimeters of mercury, *N* = number of patients, PbtO_2_ = brain tissue oxygen, PAx = pulse amplitude index (correlation between AMP and CPP), PRx = pressure reactivity index (correlation between ICP and MAP), RAC = correlation between AMP and CPP

*Only 47 patients (41 in the ICP < 15 mmHg, and 6 in the > 20 mmHg cohorts) had PbtO_2_ monitoring

### Outcome differences: ICP below 15 mmHg versus ICP above 20 mmHg

**Fig. 2:**
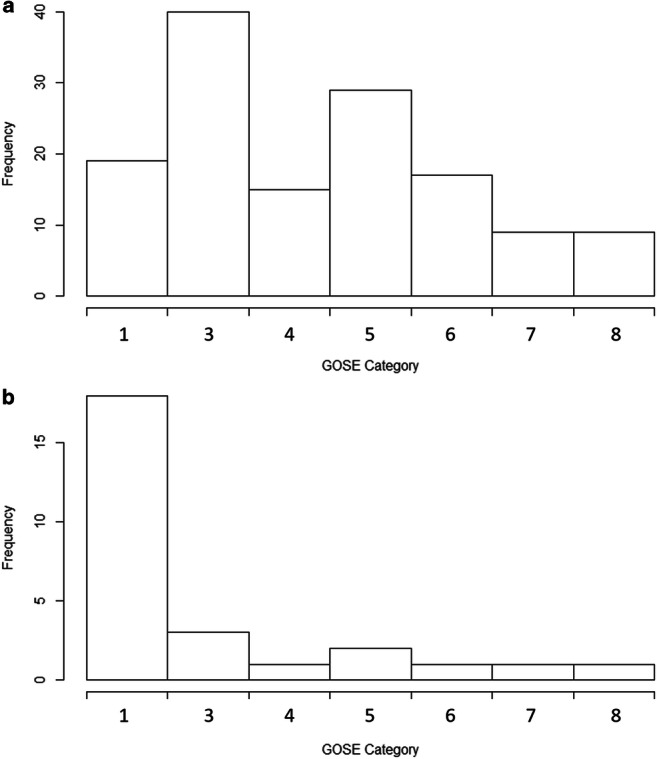
Histogram of 6-month GOSE–ICP below 15 mmHg (**a**) and ICP above 20 mmHg cohort (**b**). Frequency = number of patients, GOSE = Glasgow Outcome Score Extended. **a** Mean ICP < 15 mmHg cohort. **b** Mean ICP > 20 mmHg cohort

Comparing 6-month outcome between the two ICP extreme groups, there was a clear association between persistent ICP elevations (i.e., ICP above 20 mmHg) and worse outcome. Figure 2 provides a histogram of the GOSE scores for both the ICP below 15 mmHg (Fig. 2a) and the ICP above 20 mmHg (Fig. 2b) groups. This figure highlights the disparity of outcomes based on this ICP dichotomization. Similarly, evaluating differences in dichotomized GOSE outcomes at 6 months, there is a statistically higher mortality (*p* < 0.0001) and unfavorable outcome (*p* = 0.014), using *χ*^2^ testing. The independent association of ICP with outcome has previously been explored in the CENTER-TBI HR-ICU sub-study cohort [33]. To confirm that this relationship holds true in the cohort described in this manuscript, we evaluated the association between mean ICP and dichotomized outcomes using multivariable logistic regression models, adjusting for baseline admission characteristics (see Methods for list of characteristics). For the association with mortality, ICP maintained an independent association (*p* = 0.0005), when adjusting for baseline admission characteristics (AUC = 0.801, 95% CI 0.716–0.884 for full model). However, for association with unfavorable outcome, mean ICP failed to maintain significance (*p* = 0.126) when adjusting for baseline admission characteristics (AUC = 0.678, 95% CI 0.584–0.762, for full model).

## Discussion

Using the CENTER-TBI HR-ICU sub-study data set, we have been able to provide some prospective multi-center validation for previously described cerebral physiologic responses to ICP extremes. Some important aspects deserve highlighting.

First, sustained elevations in ICP in the current era of critical care management of TBI patients are rare. Out of the 225 non-EVD ICP monitored patients from the HR-ICU cohort, only 28 had sustained elevations in ICP to generate a 3-day mean above 20 mmHg. The vast majority (*n* = 157) had a mean ICP below 15 mmHg. This speaks to the active treatments received by these patients during the course of their ICU care and highlights the successes of aggressive care [6, 14, 16, 32].

Second, comparing those patients with mean ICP below 15 mmHg, versus above 20 mmHg, the only apparent difference in admission demographics/characteristics were the admission CT grade, both Marshall and Rotterdam. There was no difference in other admission factors between these two cohorts. This emphasizes the association between primary injury pattern/severity and impaired cerebral physiology [17, 30, 35, 36]. Such findings corroborate previous retrospective results.

Third, 6-month outcomes were worse in those with a 3-day mean ICP above 20 mmHg. In particular, the association with 6-month mortality was much stronger than that of unfavorable outcome. This has been described previously, where ICP appears to be a stronger predictor of mortality over functional outcome post-TBI [6, 24]. This held true in multi-variable logistic regression analysis, adjusting for baseline admission characteristics. Such worse outcomes are likely driven not only by ICP alone but by a myriad of cerebral physiologic derangements. It must be acknowledged that increased ICP may not in itself lead to poor outcome in TBI but may just be an indicator of overall injury severity and globally impaired homeostasis, which is difficult to correct and may in turn drive poor outcomes. This is further supported by the percentage of time with CPP below 60 mmHg and above 70 mmHg, where the ICP > 20 mmHg cohort had significantly higher proportion of time with CPP below 60 mmHg. Conversely, in the normal ICP group, only slightly less than a half of patients had favorable outcome. It means that apart from intracranial hypertension, other factors may be responsible for worse outcome. One of them, as identified previously, is deterioration in cerebrovascular reactivity [12, 14, 24, 33]. Finally, if deranged homeostasis is the true driver of poor outcomes in TBI, the concept of therapeutic fatigue becomes relevant, where persistent ICP elevations despite active attempts to treat may lead to situations where the treating team is more prone to de-escalating care over concerns of futility. Such attitudes toward certain physiologic responses may influence the statistical relationships seen and are difficult to account for.

Fourth, brain tissue oxygenation trended toward lower values in those with elevated ICP. This was seen in percentage of time with PbtO_2_ below 20 mmHg, where there was a trend toward lower mean and higher percentage of time below threshold in the ICP above 20 mmHg group. Similarly, evaluating the mean hourly dose of PbtO_2_ below 20 mmHg, there was a statistically significant high mean hourly dose in the ICP above 20 mmHg group. This highlights previously described relationships between ICP and extracellular oxygen. Such findings, as well as literature supporting the association of low PbtO_2_ and normal ICP with poor outcome in TBI, have sparked the phase II and ongoing phase III trials for PbtO_2_-directed therapy in TBI [7, 22]. It must be emphasized, however, that these findings do not suggest a specific directional causality between ICP and PbtO_2_, but only provide evidence in support of an association. Much further multi-center work is required, investigating the temporal causal relationships between various aspects of cerebral physiology.

Fifth, ICP elevations are associated with worse cerebrovascular reactivity. This was seen measuring cerebrovascular reactivity using PRx, PAx, and RAC. Regardless of which index was assessed, there was a statistically significant high mean, percentage of time above threshold, and mean hourly dose above threshold in the ICP above 20 mmHg group. This held true for every index threshold tested for PRx, PAx, and RAC. This finding validates previous retrospective single-center findings that ICP elevations are associated with worse cerebrovascular reactivity [1, 10]. However, another interesting finding in this study is that those with ICP below 15 mmHg still had a median percentage of time with PRx above 0 and + 0.25 of 47.8 (IQR 22.6–64.3) and 23.7 (IQR 14.0–38.0), respectively. This suggests that despite ICP control, moderate/severe TBI patients still display significant time periods in the first 3 days of care with impaired cerebrovascular reactivity. Recent work has highlighted the association between impaired cerebrovascular reactivity and outcome, as well as its apparent independence to current BTF-based therapeutic interventions for TBI [14, 32]. The findings in this study indicate the need for cerebrovascular reactivity–based interventions, regardless of ICP control, so as to prevent ongoing secondary injury related to impaired cerebral autoregulation. Such work is the focus of various collaborative efforts globally [4, 20, 37]. However, it must be emphasized that the results here are merely associations, and do not prove causality in the relationships between ICP elevations and impaired cerebrovascular reactivity. Further investigation into the temporal profiles of cerebral physiology responses are required.

Finally, cerebral compensatory reserve, as measured through RAP and its integrated AUC above 0, was found to be worse in those with ICP above 20 mmHg. Given that the relationship between RAP and ICP is an inverse U relationship, we utilized the integrated AUC to characterize the insult burden of RAP over time. As ICP remains elevated, RAP transitions from + 1 (impaired compensatory reserve) toward 0 and eventually a negative number (indicating severe intracranial hypertension and associated collapse of cerebral arterioles, disturbing integrity of cerebral blood flow). The integrated AUC above 0 was found to be significantly lower (*p* = 0.047) in the ICP > 20 mmHg cohort, indicating worse overall compensatory reserve. These findings are the first multi-center international findings to validate previous single-center retrospective work on RAP in TBI [5]. However, again, these findings do not support a directional causal relationship between ICP and RAP, but merely an association. Further investigation into the use of continuously updating RAP monitoring in TBI is required.

### Limitations

Despite the interesting results found, there are some significant limitations of this study which deserve highlighting. First, this is a multi-center cohort of TBI patients receiving active ICP- and CPP-directed therapy. Thus, the impact of various interventions on recorded cerebral physiology cannot be accounted for. Such interventions will have impacted the physiology recorded. As such, the physiology does not represent the natural history of moderate/severe TBI.

Second, despite being multi-center and international in nature, our data set is still relatively small, with only 157 patients in the below 15 mmHg and 28 in the above 20 mmHg cohorts, respectively. As such, despite providing some validation for previous single-center retrospective works, the results here should still be considered exploratory in nature. Thus, the reported findings between different cerebral physiologic measures and ICP merely represent associations and do not provide evidence supporting any directional causal relationship at this time. Future work in the field of multi-modal cerebral physiologic monitoring necessitates widespread multi-center data collection initiatives, to generate larger data sets.

Third, given this was an exploratory project with a relatively small data set, we did not correct for multiple comparisons. Some of the described “significant” results would not have been significant if such correction were taken into account, particularly PbtO_2_ and RAP AUC above 0. Thus, the reported statistically significant results need to be interpreted with caution and as exploratory at this time, as they represent preliminary associations only.

Finally, given this is one of the first multi-center prospective data sets with high-frequency physiology to analyze, we focused primarily on descriptive analysis of the difference between extreme ICP cohorts. The complex interaction between various multi-modal monitoring cerebral physiology (raw or derived) cannot be commented on here. Such work requires extensive data sets with numerous invasive/non-invasive monitors simultaneously recording in every patient. Only then can statements be made regarding the in vivo temporal relationship between various cerebral physiologic measures. The HR cohort from CENTER-TBI does not have large numbers of such multi-modal data. This is highlighted by the relatively small number of PbtO_2_ patients in this study (*n* = 47). Therefore, as commented on previously, the reported relationships between ICP elevation and other aspects of cerebral physiology only represent associations and do not imply causality. Future investigation into the temporal and directional causal relationships between ICP and other aspects of multi-modal cerebral physiologic monitoring will require the application of advanced time-series techniques. Such work would include the use of linear/non-linear mixed modeling techniques, incorporating individual signal Box–Jenkin’s autoregressive integrative moving average (ARIMA) structures, development of multivariate vector autoregressive moving average (VARMA) models with impulse response function plots, as well as Granger causality testing [25, 27, 28]. As the focus of this study was to provide preliminary multi-center descriptions of the relationships between multi-modal cerebral physiology during ICP extremes, this advance time-series modeling is beyond the scope of this paper. Such complex work with multi-center data sets are the focus of ongoing collaborative initiatives in TBI research both in Europe [20] and Canada [4].

#### Conclusions

Extremes in ICP during the first 72 h after moderate/severe TBI are associated with significant disparities in CPP, AMP, cerebrovascular reactivity, cerebral compensatory reserve, and brain tissue oxygenation metrics. Such ICP extremes appear to be strongly related to 6-month patient outcomes, in keeping with previous literature. This work provides multi-center validation for previously described single-center retrospective results.
